# Virtual Reality Relaxation for Reducing Aggression and Emotional Distress in At‐Risk Adolescents in Israeli Residential Care: A Feasibility Randomized Clinical Trial

**DOI:** 10.1002/ab.70057

**Published:** 2026-01-05

**Authors:** Hagit Mercedes Sabo‐Brants, Barak Ariel, Brad J. Bushman

**Affiliations:** ^1^ Western Galilee College Acre Israel; ^2^ University of Cambridge Cambridge UK; ^3^ The Hebrew University of Jerusalem Jerusalem Israel; ^4^ The Ohio State University Columbus Ohio USA

**Keywords:** adolescents, aggression, emotional distress, flow, relaxation, residential care, virtual reality

## Abstract

Aggression and emotional distress are all too common in at‐risk adolescents in closed residential institutions, which often contain restrictive conditions such as overcrowding, lack of autonomy, and shifting staff–youth relationships. In war‐torn areas like Israel, these restrictive conditions are amplified with additional stressors such as the threat of injury or even death. In such settings, feasibility studies are greatly needed to investigate how to reduce aggression and emotional distress in vulnerable adolescents. This feasibility randomized clinical trial evaluated the effectiveness of a virtual reality (VR)‐based relaxation intervention in decreasing self‐reported aggression tendencies and emotional distress and increasing positive psychological states among at‐risk Israeli adolescents in residential care. Fifty‐two participants from different residential facilities were randomly assigned to a treatment group (*n* = 27), who received twice‐weekly VR relaxation sessions, or a waitlist control group (*n* = 25). Self‐report measures of aggression and emotional distress were administered at baseline, midway through the intervention (2 weeks), and at the end of the intervention (4 weeks). Mixed‐effects linear models suggest that the treatment group demonstrated clear reductions in both aggression and emotional distress across the intervention period relative to the control group (Hedges' *gs*: 0.85–1.42). In addition, immediately before and after each VR session, participants in the treatment group reported consistent increases in positive mood and flow experience (Hedges' *gs*: 0.45–0.73). These preliminary findings support the utility of brief VR relaxation practices in reducing negative emotional states and fostering well‐being among high‐risk youth in closed institutional settings.

**Trial Registration:** This study was pre‐registered on the Open Science Framework (OSF) prior to data collection. The pre‐registration is available at: https://osf.io/pr3md/.

## 1 Introduction

Many young offenders and at‐risk youth have experienced adverse life circumstances, including emotional and behavioral maladjustments, familial instability, poverty, and early trauma (Hale and Viner 2016). Such vulnerabilities often result in placement within custodial institutions, where the restrictive conditions and living with youths with similar issues can exacerbate the underlying psychological and emotional issues, including aggression and crime (Janssen‐de Ruijter et al. 2017; Layne et al. 2014). Exposure to ethnic‐political conflict further compounds these vulnerabilities, particularly in conflict regions such as Israel, where longitudinal evidence demonstrates that such exposure increases emotional distress, aggressive behavior, and intergroup hostility (Dubow et al. 2009; Dubow, Boxer, et al. 2012; Dubow, Huesmann, et al. 2012; Huesmann et al. 2017; Niwa et al. 2016). Although exposure to violence is only one of many risk factors for aggression, it is an important one.

Evidence‐based interventions—such as cognitive behavioral therapy, prolonged exposure, cognitive processing therapy, and mindfulness‐based approaches—can sometimes mitigate behavioral and neurobiological sequelae of such adverse childhood experiences in care settings (Kirlic et al. 2020; Tao et al. 2021). Mindfulness‐based interventions (MBIs) are among the most promising and have been shown to reliably reduce anger, aggression, impulsivity, and anxiety by enhancing emotion regulation, attentional control, and cognitive reappraisal (Evans‐Chase 2013; Kjærvik and Bushman 2024; Simpson et al. 2018; Zoogman et al. 2015). However, traditional mindfulness formats, such as mindfulness and relaxation techniques, often face low adherence and engagement challenges among adolescents (Mettler et al. 2023; Wieczorek et al. 2024).

One possible way to engage with youth is through computer‐aided apparatuses. Specifically, virtual reality (VR) platforms can embed therapeutic or self‐regulation exercises within immersive, interactive environments that help address the low engagement problem (S. Zhang et al. 2023; Arpaia et al. 2022; Wieczorek et al. 2024). Clinically, research has demonstrated that VR‐Mindfulness, for example, can enhance emotion regulation, mood, and behavioral manifestations more effectively than traditional approaches (Seabrook et al. 2020; Ma et al. 2023; Wieczorek et al. 2024). Electroencephalogram (EEG) evidence of increased alpha and reduced beta activity further validated its therapeutic potential (Tauscher et al. 2019; Yang et al. 2019; Y. Zhang et al. 2021; Zheng et al. 2024). However, most VR interventions target hospitalized or adult samples. A recent scoping review of VR interventions in forensic behavioral contexts (including all types of VR applications) identified only 25 studies in total, almost exclusively with adults, and only two small‐scale projects involving youth (Mertens and van Gelder 2025)—thus leaving a gap in residential and juvenile‐justice contexts (Antonovics et al. 2024). To fill this void, the present study reports on the utility of “FloatVR,” a VR‐assisted relaxation treatment explicitly designed to mitigate aggression and emotional distress among at‐risk adolescents in residential care.

In the present study, we used FloatVR, a brief VR‐based calming protocol that offers an immersive relaxation environment without guided meditation or breath‐focused instructions. As such, it corresponds more closely to VR‐assisted relaxation than to traditional VR‐MBIs. Relaxation‐based approaches activate key regulatory mechanisms such as arousal reduction, attentional anchoring, and emotional modulation, which are directly relevant to the theoretical frameworks guiding this trial and have demonstrated effectiveness in reducing distress and anxiety among adolescents (Hamdani et al. 2022). The intervention was also designed as a low‐burden, routine‐compatible tool that can be implemented by nonclinical staff within daily schedules in closed residential care, rather than as a high‐burden full‐scale VR psychotherapy program. In line with longitudinal research on youth in conflict‐affected regions, the present trial therefore conceptualizes emotional distress and aggression as interrelated outcomes, whereby elevated distress and arousal can undermine self‐regulation and increase the likelihood of aggressive responding (Boxer et al. 2013; Dubow et al. 2009; Dubow, Boxer, et al. 2012; Dubow, Huesmann, et al. 2012; Dubow et al. 2019; Huesmann et al. 2017; Niwa et al. 2016).

### 1.1 Theoretical Framework

Three complementary theoretical frameworks underpin this randomized clinical trial (RCT): the General Aggression Model (GAM; Anderson and Bushman 2002; 2018; Anderson et al. 2017), Presence Theory (Slater and Wilbur 1997), and Flow Theory (Csikszentmihalyi 2000; Nakamura and Csikszentmihalyi 2002). Together, they clarify why a VR‐assisted relaxation protocol might decrease aggression and emotional distress, and how its design can be optimized for therapeutic impact. Virtual environments can be designed to replicate real‐world scenarios and contexts with a degree of interactivity that can be manipulated based on these theoretical models.

The General Aggression Model offers a comprehensive meta‐theoretical framework for understanding how individual and situational factors interact to produce aggressive behavior. It posits that distal or personological inputs (e.g., early childhood adversity, development of aggressive cognitive schemas, and dispositional traits like chronic anger) shape one's baseline propensity for aggression. These distal factors operate alongside proximate or situational inputs (e.g., exposure to violent media, provocation, and social exclusion) or direct experiences like witnessing armed conflict (ELEM 2024; Levi‐Belz et al. 2024; Groweiss et al. 2024) serve as immediate triggers. Both sets of inputs feed into internal states (cognitive, affective, and arousal‐related) that are processed through appraisal and decision‐making mechanisms. Initial judgment of certain situations, often rapid and automatic, can result in impulsive, aggressive actions if left unregulated, whereas more deliberate reappraisals may modulate or inhibit these responses. In the context of the present intervention, the low‐threat VR environment is expected to interrupt rapid threat appraisals and thereby create cognitive space for reappraisal.

Presence Theory examines the psychological experience of “being there” in a mediated environment. Heightened spatial presence, created by immersive realism, vivid sensory cues, and interactivity, can strengthen the user's sense of inhabiting a mediated space (see also Kuhne et al. 2023). This immersive state influences attentional allocation, perceptual realism, and emotional engagement. In contexts involving potentially aggressive stimuli, a stronger sense of presence can amplify affective responses and prolong the engagement of self‐regulatory processes, potentially intensifying or mitigating aggression depending on the individual's predispositions and situational framing (Lull and Bushman 2016). This form of presence derives specifically from system immersion (Nilsson et al. 2016). In this intervention, spatial presence serves a regulatory function by stabilizing perception and reducing external distractions, thereby supporting the conditions needed for emotional modulation rather than reactive responding.

Flow Theory describes an optimal psychological state characterized by deep concentration, intrinsic enjoyment, and altered temporal perception, arising when an individual's perceived skills are in balance with the perceived challenge of the task at hand(Peifer et al. 2022). In a flow state, attentional resources are fully invested in the activity, self‐consciousness is diminished, and feedback loops between action and outcome become immediate and clear. Within the present study's scope, flow can facilitate sustained engagement in activities requiring emotional self‐regulation, as the balance between challenge and skill may help buffer against impulsive, aggression‐related responses by maintaining focus on task‐related goals rather than on provocative stimuli. Flow therefore functions here as a therapeutic mechanism by sustaining attentional absorption and reducing self‐focused or threat‐oriented processing, which can help regulate aggression‐related impulses.

To further clarify how these theoretical mechanisms operate in VR environments, it is essential to distinguish between different forms of immersion that can give rise to distinct modes of “being there.” A seminal contribution to the study of immersion clarifies that virtual environments afford multiple forms of “being there” (Nilsson et al. 2016). Specifically, immersion can be conceptualized as consisting of (a) system immersion, which produces spatial presence through sensory fidelity; (b) challenge‐based immersion, which underlies flow and reflects attentional absorption in an activity; and (c) narrative or content‐based immersion, which reflects engagement with the emotional tone and meaning of the virtual scene. These distinct mechanisms are all relevant to VR‐based relaxation interventions and operate differently from provocation‐driven or threat‐related immersion. In the present intervention, these immersive properties are deliberately harnessed to support calming, non‐provocative experiences rather than to elicit threat or conflict, aligning immersion with regulatory rather than reactive functions.

### 1.2 VR‐Based Interventions

Thus, these theoretical frameworks address complementary aspects of how VR can reduce aggression and emotional distress. The General Aggression Model explains how personal and situational factors interact to produce aggressive responses, providing the behavioral foundation. Presence Theory accounts for how VR's immersive qualities can heighten emotional and cognitive engagement, amplifying or dampening aggression‐related processes identified in the GAM. Flow Theory adds a motivational lens, showing how balancing challenge and skill can sustain focus on adaptive regulation rather than impulsive reactions. For aggression‐focused interventions, VR allows participants a controlled and safe space, where behavioral, cognitive, and emotional responses can be elicited, monitored, and guided toward adaptive regulation (Jin 2011; Smeijers et al. 2021). This combination of immersion, realism, and control makes VR uniquely suited to testing and training self‐regulatory skills in ways that traditional role‐play or discussion‐based approaches cannot match.

A recent meta‐analysis (Roncero et al. 2025) synthesizing 11 studies on VR interventions targeting aggression‐related constructs in predominantly adult forensic and clinical samples (*N* = 479; 88% male) found statistically significant pre–post reductions in observer‐rated aggression (Hedges' *g* = −0.27), self‐reported aggression (*g* = −0.47), anger (*g* = −0.74), and impulsiveness (*g* = −0.47), with a pooled controlled effect size of *g* = −1.05 favoring VR. These findings suggest that immersive VR can reduce aggression‐related outcomes in adults. However, the evidence base remains narrow in scope and limited in methodological quality. An exception is the multicenter VRAPT trial (Klein Tuente et al. 2020), which demonstrated the feasibility of a large‐scale VR‐based aggression prevention program among 128 forensic inpatients. However, like most VR interventions, VRAPT adapted an existing therapeutic protocol into VR and was delivered over multiple sessions by trained clinicians, which can be quite costly.

VR is commonly used to translate established psychosocial models, such as Cognitive Behavior Therapy, Aggression Replacement Training, or Social Information Processing, into immersive formats. These adaptations typically preserve the structure, duration, and therapist involvement of the original treatment, rather than leveraging mechanisms that are intrinsic or unique to VR itself (e.g., spatial presence and flow). Even when targeting youth populations, such as in the Your Skills program (Alsem et al. 2023), VR functions as an adjunct to a therapist‐led cognitive‐behavioral framework, not as an independent intervention modality. A parallel and underexplored line of research has focused on brief, stand‐alone VR relaxation tools that operate without structured therapeutic content. These interventions, often referred to as VR‐assisted relaxation, rely on immersive naturalistic environments (e.g., forests, beaches, underwater scenes) to elicit calm and reduce negative effects (Riches et al. 2021; Ahn et al. 2025). They are typically delivered in single or short sessions and do not rely on clinical guidance or therapeutic instructions.

Although randomized controlled trials exist on non‐guided immersive VR relaxation, none have tested their effects on aggression‐related outcomes among high‐risk adolescents. Nonetheless, evidence from pediatric medical contexts consistently shows that brief VR interventions can reduce acute emotional distress among children and adolescents. For example, VR appears to be a valuable strategy for reducing pain, fear, and anxiety during needle‐related procedures (Cáceres‐Matos et al. 2024; Wong and Choi 2023), anesthesia induction compliance (Ryu et al. 2018), circumcision surgery (Luo et al. 2023), and among pediatric oncology outpatients (Reitze et al. 2024).

Taken together, existing research points to two distinct lines of development: (1) structured, therapist‐led VR interventions that translate established psychosocial treatments, including mindfulness‐based protocols, into immersive formats; and (2) brief, standalone, non‐guided VR relaxation environments that are designed primarily to reduce stress in low‐risk adult samples. To date, no randomized trial has tested whether a brief, stand‐alone, noninstructional VR experience can reduce aggression and emotional distress among adolescents in closed residential care. The present study addresses this gap by evaluating a minimalist VR relaxation tool that is not derived from an existing therapeutic protocol, does not involve therapist guidance, and does not include any instructional, meditative, or narrative components, relying instead solely on immersive sensory input to support emotional regulation.

In line with Nilsson et al. (2016), the present intervention engages three forms of immersion: system immersion that provides perceptual stability; challenge‐based immersion that supports flow; and sensory‐affective immersion elicited by calming visual environments. None of these forms involve provocation‐related cues, and together they facilitate a mindful, non‐reactive experience of “being there.”

## 2 Methods

### 2.1 Study Design

To test these hypotheses, we implemented a waiting list RCT, with FloatVR treatment—using two “worlds” in multiple 7‐min sessions—among Israeli adolescents in closed and secure residential care. Participants were assessed at three time points: baseline (T1), mid‐intervention after 2 weeks (T2), and at 4 weeks post‐randomization (T3).

### 2.2 Participants

A power analysis was conducted to determine the number of participants needed to detect a large effect size (i.e., *g* = 0.80) with power = 0.80 at the 0.05 two‐sided significance level (Cohen 1988). We assumed a large effect size based on previous meta‐analyses that have shown large effect sizes for VR manipulations across multiple domains (e.g., Carl et al. 2019; Goudman et al. 2022; Ozturk and Toruner 2023; Corrigan et al. 2023). The effect size was Hedges' *g*, which corrects for small‐sample bias (Borenstein et al. 2011). This power analysis revealed that 52 participants were needed.

Participants (*N* = 52; age 14–19, *M* = 16.4, SD = 1.53; 80.8% male) were recruited from three secure Israeli residential facilities (one Arab‐male, one Jewish‐male, and one mixed‐gender). Details are provided in Supporting Information S1.

### 2.3 Procedure

Informed consent was secured from the legal guardians of participants, and assent was obtained from the participants themselves. All participants completed three assessments (i.e., T1–T3). Participants in the treatment group engaged in 7‐min VR sessions of VR‐based mindfulness using the FloatVR application (approximately twice per week). Treatment participants used the FloatVR application to immerse themselves in tranquil, visually engaging environments accompanied by ambient music and soothing visual stimuli. The experience did not include guided voiceovers or meditation instructions, enabling participants to engage in self‐directed relaxation within the virtual space. Each session lasted 7 min, and scheduling was adapted based on availability and logistical constraints. Data were collected from both treatment and control participants at each juncture (i.e., T1–T3), whereas treatment participants were also provided flow and mood ratings immediately before and after three VR sessions. A planned 2‐week follow‐up (T4) was canceled due to a nationwide missile attack, making T3 the final time point. Trained research assistants conducted data collection and entry (see Supporting Information S1).

### 2.4 Measures

Measurements were collected before and after three VR sessions (six data points per participant) (see Supporting Information S1 for full scale descriptions, translations, and psychometric details). Aggression was measured using the 29‐item (e.g., “Given enough provocation, I may hit another person”) Aggression Questionnaire (Buss and Perry 1992). Participants rated each item on a 7‐point scale ranging from 1 (*not at all*) to 7 (*very much*). Reverse‐scored items were recorded. Reliability of the total (non‐language‐split) scale was strong across all three assessment points (McDonald Ω's ranged from 0.88 to 0.93; Cronbach *α*'s ranged from 0.87 to 0.90).

Emotional distress was assessed using the 21‐item (e.g., “I had difficulty experiencing any positive feelings”) version of the Depression Anxiety Stress Scales (DASS‐21; Lovibond and Lovibond 1995). Participants were asked to rate how much each statement applied to them over the past week on a 4‐point scale ranging from 0 (*did not apply at all*) to 3 (*applied to a great extent*). Reliability of the total (non‐language‐split) scale was strong across all three assessment points (McDonald Ω's ranged from 0.89 to 0.95; Cronbach *α*'s ranged from 0.89 to 0.95).

Within the VR treatment group, flow was assessed using the 8‐item (e.g., “my thoughts/activities run fluidly and smoothly”*)* Flow Short Scale (FSS; Rheinberg et al. 2023) (McDonald Ω's ranged from 0.75 to 0.89; Cronbach *α*'s ranged from 0.76 to 0.89). Mood was also assessed using an adapted version of the State‐Trait Personality Inventory (STPI; Spielberger 1995; adapted by Ben‐Zur and Zeidner 1987). Due to time constraints and participant burden, a single representative item was used for each emotional indicator, formulated as a cluster of related emotional descriptors. Participants rated the extent to which they currently experienced each of seven mood states on a 5‐point Likert scale ranging from 0 (*not at all*) to 4 (*very much*). A composite positive mood index was constructed based on five non‐neutral items: “Happy,” “Calm,” and the reverse‐coded items “Anger,” “Depressed Mood,” and “Panic.” The items “Tired” and “Vigor” were excluded, as they were considered relatively neutral in affective tone(McDonald's *ω*'s ranged from 0.63 to 0.84; Cronbach *α*'s ranged from 0.54 to 0.80).

### 2.5 Statistical Analysis

All data were screened for data‐entry errors and for the presence of potential univariate or multivariate outliers; no cases were removed. We examined patterns of missingness—5.8% (emotional distress) and 7.7% (aggression) at T1; 23.1% (emotional distress) and 25.0% (aggression) at T2; and 23.1% for both measures at T3—and found no systematic bias: Little (1988) MCAR test indicated missing completely at random (*χ* ^2^ = 158.33, df = 209, *p* = 0.996), justifying full information maximum likelihood (FIML) in subsequent models (Gabrio et al. 2022). We inspected normality of residuals via *Q*–*Q* plots and Shapiro–Wilk tests (Shapiro and Wilk 1965) and assessed homogeneity of variances with Levene's test (Gastwirth et al. 2009); no meaningful violations were found. Multicollinearity among predictors was evaluated using variance inflation factors (VIF < 2 for all variables).

Inferential analyses were conducted in SPSS v.27 and R v.4.2. Primary outcomes were tested using linear mixed‐effects models (lme4 package), with Time (T1–T3) and Group (treatment vs. control) as fixed effects, their interaction as the effect of interest, Participant ID as a random intercept, and Facility as a covariate. We used restricted maximum likelihood estimation and Satterthwaite's (Kuznetsova et al. 2017) method for denominator degrees of freedom. Significant Time × Group interactions were followed by pairwise comparisons of estimated marginal means (emmeans package), with Bonferroni correction to control for multiple measurements. All tests were two‐tailed with *α* = 0.05.

Finally, exploratory session‐level effects on flow and mood (which were measured only in the treatment arm) were examined using paired‐samples *t*‐tests comparing pre‐ and post‐session scores for each of the three monitored sessions separately; effect sizes (Hedges' *g*) are reported. Full analytic scripts, assumption checks, and detailed output tables are provided in Supporting Information S1.

## 3 Results

### 3.1 Effects of the VR‐Based Mindfulness on Aggression

The analysis revealed a significant main effect of Time, *F*(2, 75.84) = 5.29, *p* = 0.007, and a significant main effect of Group, *F*(1, 47.49) = 12.02, *p* = 0.001. Most importantly, the Time × Group interaction was also significant, *F*(2, 75.67) = 5.97, *p* = 0.004, indicating differential changes in aggression across time between groups. No significant effect was found for Facility (*p* > 0.31), suggesting consistency across institutions (see Table SM1).

**Figure 1:**
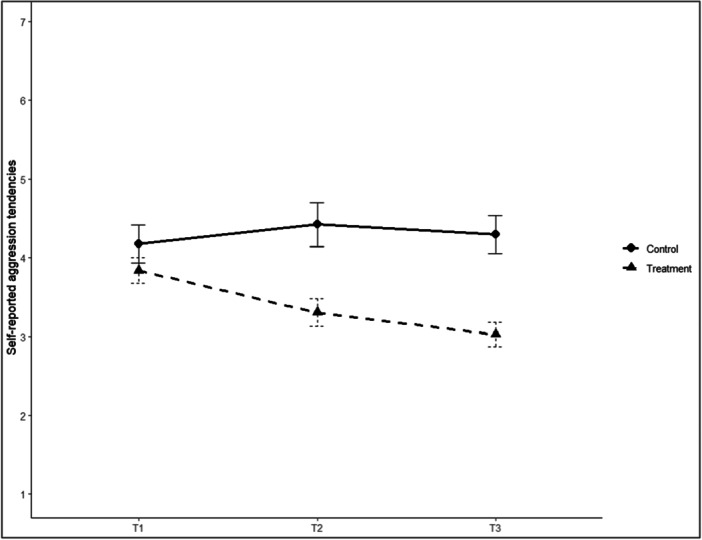
Aggression levels for control and treatment groups at three time points. Capped vertical bars denote standard errors. *Note:* T1 = pre‐treatment; T2 = 2 weeks post‐randomization; and T3 = 4 weeks post‐randomization.

Figure 1 illustrates the trajectories of aggression scores for the control and treatment groups across three assessment points (T1–T3). At baseline (T1), the mean aggression score was 4.17 (SD = 1.13) in the control group and 3.84 (SD = 0.84) in the treatment group, indicating moderately high levels of self‐reported aggression in both groups. The two groups did not differ, After 2 weeks (T2), mean aggression remained high in the control group (*M* = 4.42, SD = 1.15), while the treatment group showed a notable reduction (*M* = 3.30, SD = 0.81). After 4 weeks (T3), the control group's aggression scores remained elevated (*M* = 4.29, SD = 1.02), whereas the treatment group continued to decline (*M* = 3.03, SD = 0.74), reflecting a meaningful reduction over time.

The statistical models confirm these descriptive results. At baseline (T1), no significant difference in aggression was found between the treatment and control groups (*p* > 0.16, *g* = 0.33, 95% CI [−0.24, 0.91]). After 2 weeks (T2), the treatment group showed statistically significantly lower aggression scores than the control group (*p* < 0.001, *g* = 1.13, 95% CI [0.44–1.82]). This difference remained significant and increased after 4 weeks (T3) (*p* < 0.001, *g* = 1.42, 95% CI [0.71–2.12]).

Furthermore, within‐group comparisons indicated that aggression in the treatment group significantly decreased from T1 to T2 (*p* = 0.013) and from T1 to T3 (*p* < 0.001). In contrast, the additional decrease from T2 to T3 was nonsignificant (*p* = 0.69). No significant changes were observed within the control group across time (*p* > 0.13).

### 3.2 Emotional Distress

The analysis revealed a significant main effect of Time (*F*(2, 78.93) = 7.58, *p* < 0.001), and a significant main effect of Group (*F*(1, 47.30) = 10.51, *p* = 0.002). The Time × Group interaction was also significant (*F*(2, 78.18) = 4.40, *p* = 0.016), indicating different trajectories of emotional distress between the treatment and control groups over time. Again, no significant effect was found for Facility (*p* > 0.28), suggesting consistency across institutions (see Table SM2).

**Figure 2:**
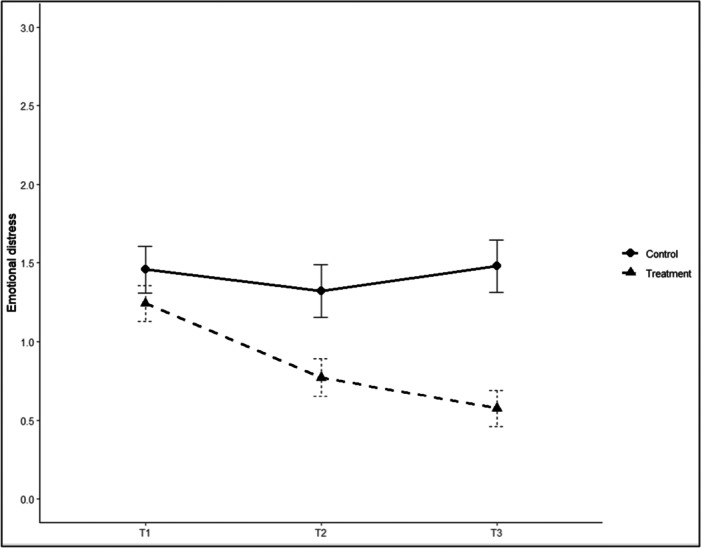
Emotional distress levels for control and treatment groups at three time points. Capped vertical bars denote standard errors. *Note:* T1 = pre‐treatment; T2 = 2 weeks post‐randomization; and T3 = 4 weeks post‐randomization.

Figure 2 illustrates the trajectories of emotional distress scores for the control and treatment groups across three assessment points (T1–T3). At baseline (T1), the control group reported a mean emotional distress score of 1.46 (SD = 0.69), whereas the treatment group's mean was 1.24 (SD = 0.59). After 2 weeks (T2), the control group showed a slight decrease to a mean of 1.32 (SD = 0.71), while the treatment group's mean dropped more markedly to 0.77 (SD = 0.56). After 4 weeks (T3), the control group's mean returned to 1.48 (SD = 0.71), whereas the treatment group's mean fell further to 0.58 (SD = 0.54).

Pairwise comparisons clarify the nature of this interaction. While at baseline (T1), no significant difference in emotional distress was observed between the groups (*p* = 0.237, *g* = 0.33, 95% CI [−0.24, 0.90]), after 2 weeks (T2), the treatment group reported significantly lower distress levels than the control group (*p* = 0.012, *g* = 0.85, 95% CI [0.20–1.51]). The effect appears to intensify further after 4 weeks (T3), with a larger effect size relative to control conditions (*p* < 0.001, *g* = 1.42, 95% CI [0.72–2.13]).

Within‐group comparisons showed that emotional distress scores in the treatment group decreased significantly from T1 to T2 (*p* = 0.002) and from T1 to T3 (*p* < 0.001), but the decrease from T2 to T3 was nonsignificant (*p* = 0.76). In comparison, no significant changes were observed within the control group across time (*p* > 0.50).

### 3.3 Exploratory Analyses: Pre–Post Effects of VR Mindfulness on Flow and Mood

In the treatment group, paired‐samples *t*‐tests compared mean scores immediately before and after each VR mindfulness session on Flow and Mood scores. In terms of Flow, results showed a robust increase at Session 1 (Δ = 0.98, *t*(24) = 3.76, *p* < 0.001, *g* = 0.69, 95% CI [0.28–1.10]), at Session 2 (*Δ* = 0.97, *t*(24) = 2.72, *p* = 0.012, *g* = 0.65, 95% CI [0.12–1.18]), and at Session 3 (*Δ* = 0.61, *t*(21) = 2.17, *p* = 0.042, *g* = 0.53, 95% CI [0.00–1.05]).

In terms of Mood, results indicated statistically significant improvements across all three sessions. In Session 1 (*n* = 25), mood scores increased significantly (*t*(24) = 2.28, *p* = 0.032, *g* = 0.44, 95% CI [0.04–0.83]). In Session 2 (*n* = 26), the improvement was more pronounced, *t*(25) = 3.03, *p* = 0.006, *g* = 0.57, 95% CI [0.17–0.98]. Session 3 (*n* = 22) also showed a significant increase in mood, *t*(21) = 3.19, *p* = 0.004, *g* = 0.65, 95% CI [0.20–1.10]. These findings demonstrate a consistent and growing pattern of mood enhancement following each VR session, suggesting that repeated exposure to the intervention may contribute to cumulative emotional benefits.

## 4 Discussion

This randomized clinical trial evaluated the effects of using a VR relaxation‐based intervention on adolescents in secure residential facilities in Israel. Consistent with our hypotheses, the intervention produced favorable improvements for both self‐reported aggression tendencies and emotional distress scores. Treatment participants demonstrated a significant reduction in self‐reported aggression tendencies over time, with between‐group differences emerging at 2 weeks (T2) and strengthening by 4 weeks (T3). In contrast, control participants showed no significant change over time.

Similarly, emotional distress in the treatment group declined markedly over time, with between‐group differences emerging at T2 and strengthening by T3. Compared to the control group, the VR group showed significantly lower levels of aggression and emotional distress at both T2 and T3, indicating robust treatment effects over multiple measurement points.

In addition to reductions in self‐reported aggression tendencies and emotional distress, the VR‐based relaxation intervention produced consistent within‐session improvements in both flow and mood. These experiential shifts may reflect more immersed presence during the virtual sessions, through mechanisms such as challenge‐based and narrative immersion, both of which support attentional absorption and affect regulation (Nilsson et al. 2016; Slater and Wilbur 1997; Csikszentmihalyi 2000; Nakamura and Csikszentmihalyi 2002). Self‐reported flow showed significant effects, particularly during the first session, indicating that participants were able to enter a focused state even in the absence of guidance. Similarly, mood improved significantly after each session, with increasing effect sizes across sessions, suggesting not only immediate emotional benefit but also a possible cumulative strengthening of mood‐related gains. It seems that the intervention induces transient yet meaningful affective and cognitive shifts during each exposure, which may contribute to the broader reductions observed in aggression and emotional distress. However, more research is needed.

In this context, the calming VR environment likely facilitates reappraisal by briefly suspending automatic threat‐oriented appraisals and allowing participants to redirect attention toward neutral or soothing sensory cues. In parallel, the increases in flow observed across sessions suggest that sustained attentional absorption may have served as an additional regulatory mechanism that reduces self‐focused rumination and dampens impulsive, aggression‐related responses.

### 4.1 Theoretical Implications

By embedding relaxation exercises within immersive, low‐threat virtual environments, VR‐based relaxation appears to attenuate the rapid threat appraisal and aggressive script activation posited by GAM, allowing for real‐time cognitive reappraisal. The significant pre‐to‐post increases in flow reported across the three monitored VR sessions support the notion that immersive engagement during VR can sustain attentional focus. Within the framework of Presence Theory (Slater and Wilbur 1997), heightened spatial presence may reduce external distractions and stabilize perceptual experience. However, spatial presence is theoretically distinct from the challenge‐based immersive processes that underline flow ` (Csikszentmihalyi 1975/2000; Nakamura and Csikszentmihalyi 2002) and in the immersion taxonomy proposed by Nilsson et al. (2016). Therefore, spatial presence alone does not account for the flow effects observed in this study. Although empirical work (Chen et al. 2022a, 2022b; Schutte and Malouff 2023) shows that mindfulness and flow can co‐occur, the present study did not assess mindfulness and cannot draw conclusions about this relationship.

### 4.2 Practical Implications

Although this was a small‐scale feasibility trial, the findings have several practical implications for educational, public health, and residential‐care systems. The consistent reductions in aggression and emotional distress, along with within‐session improvements in mood and flow, indicate that brief VR‐based calming practices can be feasibly integrated into daily routines in closed institutions without requiring extensive staff time or specialized training. Such low‐burden tools may help reduce emotional overload and behavioral dysregulation in settings characterized by high stress, crowding, and limited autonomy. This routine level integration potential distinguishes the present approach from more intensive, therapist delivered VR treatment protocols and is central to its policy relevance.

For educational and public health agencies, these results suggest that scalable VR relaxation modules could complement existing psychosocial programs for high‐risk youth, particularly in conflict‐affected regions where emotional distress is chronically elevated. Broader dissemination would require attention to cultural adaptation, implementation guidelines, and evaluation frameworks, but the present findings offer an initial evidence base for considering VR‐based calming environments as part of routine support in residential care.

### 4.3 Limitations

One noteworthy limitation of this RCT is its small sample size, which compromises both internal and external validity. This problem is compounded by the fact that the sample lacks sufficient diversity. Furthermore, the short‐term design of the study—as virtually all VR experiments—restricts assessment of long‐term or sustained effects, leaving unanswered whether the intervention's benefits endure over more extended follow‐up periods. Such limitations hinder robust conclusions regarding the persistence of impact and long‐term efficacy, nor can we study dose‐response relationships under controlled settings.

Another limitation is that we used a trait rather than a state measure of aggression. Thus, we cannot rule out that the changes we observed on the Aggression Questionnaire (Buss and Perry 1992) were due to other factors (e.g., shifts in participants' self‐perceptions and self‐presentation or demand effects).

In addition, this study relies on self‐reported measures, which are susceptible to biases. To enhance measurement validity and detection of genuine emotion‐regulation changes among young offenders, integrating physiological assessments, such as EEG or other neurobiological markers, is a promising avenue (Tauscher et al. 2019; Gkintoni et al. 2025). Administrative records from the care facilities would also be useful as a form of triangulation.

## 5 Conclusions

VR‐relaxation offers a scalable, engaging platform to deliver a relaxation‐based intervention for youth at risk in closed care facilities. We observed reductions in aggression and mental distress levels, as well as enhancements in flow and mood scores, relative to control participants. Despite the methodological limitations, the RCT offers encouraging evidence on the utility of VR environments in therapeutic sessions for juveniles. More research is needed, particularly with larger samples and more diverse populations.

## Funding

The authors received no specific funding for this work.

## Ethics Statement

Ethical approval for the study was obtained from the Helsinki Committee for the Use of FloatVR application in Research and from the academic institution of the principal investigator. In addition, institutional access was granted by the Israeli Ministry of Welfare and the Ministry of Education.

## Consent

Consent was obtained from all parents and guardians; assent was obtained from minors.

## Conflicts of Interest

The authors declare no conflicts of interest.

## Supporting information

Supplementary_Material_combines_R1.

## Data Availability

The data sets generated during the current study are available from the corresponding author on reasonable request.
