# Supplementary material for: Virtual Reality Relaxation for Reducing Aggression and Emotional Distress in At‐Risk Adolescents in Israeli Residential Care: A Feasibility Randomized Clinical Trial
Source: Aggress Behav. 2026 Jan 5;52(1):e70057. doi: 10.1002/ab.70057 (PMC12770816; doi:10.1002/ab.70057)
Supplement: Supplementary file 1 — Supplementary_Material_combines_R1. [file AB-52-e70057-s001.docx]

**Supplementary Material**

**Detailed Methodological Procedures**

Participants were 52 adolescents aged 14 to 19 (*M*=16.4, *SD*=1.527; 80.8% boys), with 27 randomly assigned to the treatment group and 25 to the control group. Participants were recruited from three closed educational and residential facilities for at-risk youth in Israel. Although each facility differed in population composition, all three operated within a structured rehabilitative framework that combined educational and therapeutic approaches. To ensure confidentiality, the facilities are referred to by letter.

Facility A and Facility C were all-male settings, with Facility A serving Arab primarily adolescents and Facility C serving primarily Jewish adolescents. Facility B was a mixed-gender therapeutic boarding school, also serving youth at high psychosocial risk.

In Facility A, 25 adolescents participated–representing all residents of the facility. One was excluded due to epilepsy. The remaining 24 participants were randomly assigned to treatment and control groups, and all three waves of data collection were completed.

In Facility C, 18 adolescents from two residential units (“houses”) were included in the study. The other two houses in the facility were excluded due to a population considered too complex for group-based intervention. Following exclusions due to lack of consent or dropout, eight participants remained–four in each group. One participant initially assigned to the treatment group was reassigned to the control group due to low numbers, and two additional participants dropped out (one from each group). The participant from the treatment group dropped out after one session. Cooperation with the control group in this facility proved challenging, and only two waves of data were collected despite various engagement efforts.

In Facility B, 20 participants from a single grade level were enrolled, including eight girls. Three girls were initially assigned to the treatment group, but two declined to use the VR headset for hygiene-related reasons. Although they were offered sanitary covers, they chose not to proceed, and both were reassigned to the control group prior to any data collection. This reassignment might slightly affect the random allocation process. Final group composition included five girls in the control group and three in the treatment group.

Participants were recruited from three closed educational-residential facilities operating under Israel’s Ministry of Education and Ministry of Welfare. These facilities provide structured, 24-hour care for adolescents with complex psychosocial backgrounds, including histories of family instability, exposure to violence, school disengagement, behavioral difficulties, emotional distress, and previous placement breakdowns. Placement is court-referred or welfare-referred, and youth attend on-site schooling combined with therapeutic and behavioral support programs.

Facility A is a secure residential educational village serving adolescent boys with significant behavioral, emotional, and social challenges that require an out-of-home structured setting with intensive supervision and support. Facility B provides a parallel residential structure for Arab adolescent boys with similar profiles and educational needs, within a culturally adapted environment. Facility C is a closed residential youth village serving Jewish boys and girls requiring intensive educational, emotional, and behavioral intervention in a supervised setting. All three facilities operate under regulated frameworks that include on-site education, psychological services, and rehabilitation-oriented daily routines.

Across these settings, residents are typically aged 14–19 and present with elevated levels of trauma exposure, emotional distress, and disruptive or aggressive behaviors requiring structured, supportive environments.

Participants in these residential facilities typically present with a combination of behavioral dysregulation, aggression related difficulties, chronic emotional distress, and prior exposure to multiple psychosocial stressors, including family instability, community violence, and other adverse childhood experiences. Although individual trauma histories cannot be reported for confidentiality reasons, these facilities commonly serve adolescents with elevated trauma exposure.

**Procedure**

Ethical approval for the study was obtained from the Helsinki Committee for the Use of FolatVR application in Research and from the academic institution of the principal investigator. In addition, institutional access was granted by the Israeli Ministry of Welfare and the Ministry of Education. Informed consent was secured from the legal guardians of participants and assent was obtained from the participants themselves.

Participants were randomly assigned to treatment or control groups based on name lists. In Facility B, randomization was conducted by classroom assignment on the first day of data collection, with no long-term implications due to the flexible structure of daily classes and shared living spaces.

All participants completed three assessments: baseline (T1), post-intervention (T2), and a final measurement (T3). The intervention spanned four weeks. During this period, participants in the treatment group engaged in up to eight sessions of VR-based mindfulness using the FloatVR application (approximately twice per week). Each session lasted around 7 minutes, and scheduling was adapted based on availability and logistical constraints. VR sessions were conducted in a quiet room within each facility, away from the main communal areas. On each occasion, participants in the treatment group came to the VR space approximately twice per week, were asked to sit comfortably on a sofa, and were fitted with the FloatVR headset. The research assistant controlled the initiation and termination of the visual simulation, and participants were not given any tasks, therapeutic instructions, or conversational prompts before or after the session; they were simply invited to watch the immersive scene until the session ended.

Self-report questionnaires measuring aggression, emotional distress (DASS), and mindfulness were administered at all three time points (T1–T3). Only participants in the treatment group completed additional measures of flow and state mood immediately before and after the VR sessions at three core intervention stages.

A follow-up assessment had initially been planned for two weeks after T3. However, a nationwide missile attack occurred immediately following the conclusion of the study, leading to two weeks of widespread disruptions and rendering follow-up data collection impossible. As a result, T3 served as the final time point for all analyses.

All questionnaires were administered on paper due to institutional restrictions on personal electronic devices. Trained research assistants conducted the data collection, ensuring consistency by having the same team administer both the treatment and control group assessments on the same day. During the intervention period, pairs of assistants facilitated the VR sessions, supervised by the lead researcher who was present for nearly all VR sessions and all questionnaire administrations.

Data were entered into SPSS and cross-checked by both the research coordinator and a trained assistant. Questionnaires were administered in Hebrew or Arabic, based on participants’ language preferences. While all measures had validated versions in the relevant languages, the Flow Short Scale was translated for this study but had not undergone formal validation prior to its use.

**Detailed  Measures & Psychometric Properties**

**Measures**

**Buss & Perry Aggression Scale**

Aggression was measured using a 29-item (e.g., “*Sometimes I cannot resist harming others*”) Aggression Questionnaire (Buss & Perry, 1992). Participants rated each item on a 7-point Likert scale ranging from 1 (*not at all*) to 7 (*very much*). Reverse-scored items were recoded accordingly. Subscale scores and an overall aggression score were calculated by averaging the relevant items. The scale was administered at three time points: baseline (T1), post-intervention (T2), and follow-up (T3).

Internal consistency of the Total Aggression scale was excellent across all three assessment points and language versions. At baseline (T1), Omega total ranged from 0.878 (Hebrew) to 0.897 (Arabic), with Cronbach’s α between 0.854 and 0.889 for the same subgroups, and 0.883 (Ωₜ) and 0.871 (α) for the full sample. By mid‐intervention (T2), reliability improved further—Ωₜ = 0.920 and α = 0.900 for the full sample, with similarly high values in the Hebrew (Ωₜ = 0.915; α = 0.893) and Arabic (Ωₜ = 0.938; α = 0.913) versions. At the final assessment (T3), the Total Aggression scale maintained strong consistency (full: Ωₜ = 0.908, α = 0.897; Hebrew: Ωₜ = 0.895, α = 0.881; Arabic: Ωₜ = 0.931, α = 0.906), confirming that the overall score reliably captured the construct over time.

## **Combined Aggression Questionnaire Reliability (T1, T2, T3)**

|  | Time | Ωₜ (Omega Total) | α (Cronbach’s α) |
| --- | --- | --- | --- |
| Total Aggression (Full sample) | T1 | 0.883 | 0.871 |
| Total Aggression (Hebrew) | T1 | 0.878 | 0.854 |
| Total Aggression (Arabic) | T1 | 0.897 | 0.889 |
| Total Aggression (Full) | T2 | 0.920 | 0.900 |
| Total Aggression (Hebrew) | T2 | 0.915 | 0.893 |
| Total Aggression (Arabic) | T2 | 0.938 | 0.913 |
| Total Aggression (Full) | T3 | 0.908 | 0.897 |
| Total Aggression (Hebrew) | T3 | 0.895 | 0.881 |
| Total Aggression (Arabic) | T3 | 0.931 | 0.906 |

**DASS-21**

Emotional distress was assessed using the 21-item (e.g., *"I had difficulty experiencing any positive feelings*") version of the Depression Anxiety Stress Scales (DASS-21; Lovibond & Lovibond, 1995). Participants were asked to rate how much each statement applied to them over the past week on a 4-point Likert scale ranging from 0 (*did not apply at all*) to 3 (*applied to a great extent*). A total emotional distress score was computed by summing all items. This measure was administered at all three time points in validated Hebrew and Arabic versions.

Internal consistency of the DASS-21 of the total score at each assessment point was evaluated by computing Omega total (Ωₜ) and Cronbach’s α using the omega() and alpha() functions from the psych package in R. Total reliability in the full sample was excellent, with Ωₜ values between 0.893 (T1) and 0.949 (T3) and α between 0.891 and 0.947. Sample-specific coefficients in the Hebrew subsample Ωₜ ranged from 0.939 to 0.926 (α = 0.938–0.925), and in the Arabic subsample from 0.795 to 0.968 (α = 0.687–0.966).

# **DASS-21 Reliability: Ωₜ and Cronbach’s α**

| Subscale | Time | Ωₜ (Omega Total) | α (Cronbach’s α) |
| --- | --- | --- | --- |
| Total DASS (Full) | T1 | 0.893 | 0.891 |
| Total DASS (Full) | T2 | 0.922 | 0.914 |
| Total DASS (Full) | T3 | 0.949 | 0.947 |
| Total DASS (Hebrew) | T1 | 0.939 | 0.938 |
| Total DASS (Hebrew) | T2 | 0.934 | 0.933 |
| Total DASS (Hebrew) | T3 | 0.926 | 0.925 |
| Total DASS (Arabic) | T1 | 0.795 | 0.687 |
| Total DASS (Arabic) | T2 | 0.918 | 0.897 |
| Total DASS (Arabic) | T3 | 0.968 | 0.966 |

Flow was measured in the intervention group only using an adapted 8-item (e.g., *I was deeply immersed in my own thoughts*) version of the Flow Short Scale (FSS; Rheinberg et al., 2003). The original scale includes 13 items, of which 10 assess core aspects of the flow experience (such as fluency and absorption), and 3 assess worry. In the present study, only 8 items focusing on fluency, absorption, and perceived control were used, based on previous abbreviated applications of the scale in the literature (e.g., Schüler & Brunner, 2009). Participants responded on a 7-point Likert scale ranging from 1 (*not at all*) to 7 (*very much*). The FSS was translated into Hebrew and Arabic for the current study but was not formally validated in either language. Participants in the intervention group completed the scale immediately before and after each of three VR mindfulness sessions, resulting in six data points per participant.

Affective states were assessed in the intervention group using a reduced version of the State-Trait Personality Inventory (STPI; Spielberger et al., 1979), adapted into Hebrew by Ben-Zur (1998). Due to time constraints and participant burden, a single representative item was used for each emotional indicator (e.g., tension, calmness), rated on a 5-point Likert scale ranging from 0= *not at all* to 4= *to a very great extent*. These brief assessments were collected before and after each of the three intervention sessions alongside the Flow Scale.

**Statistical Analysis**

**Missing Data Patterns and Handling**

Univariate inspection of total DASS and aggression scores showed that missing data increased across assessments: at baseline (T1), 5.8 % of DASS and 7.7 % of aggression scores were missing; by post-intervention (T2), missingness had risen to 23.1 % for DASS and 25.0 % for aggression; and at the third assessment (T3), both measures exhibited 23.1 % missing data. Little’s MCAR test (χ² = 158.33, df = 209, p = .996) indicating no systematic pattern of missingness and supporting the plausibility of MCAR or MAR assumptions.

Quantitative analyses were performed using SPSS and R. Prior to hypothesis testing, data were screened for entry errors, missing values, and adherence to statistical assumptions. Scale scores were calculated as detailed in the Measures section (for example, mean scores for aggression subscales, total scores for the DASS-21, and indices of internal consistency). Unless otherwise specified, all tests were two-tailed with an α level of .05.

To examine the intervention’s effects on aggression and emotional distress, we used mixed linear models (MLMs) with repeated measures. This method was selected for its robustness to missing data and ability to model time as a within-subject factor. Fixed effects included Time (T1, T2, T3), Group (treatment vs. control), and their interaction, with Participant ID as a random intercept. Separate models were estimated for each outcome

The primary test of interest was the Time × Group interaction, indicating differential change patterns between groups. When significant, pairwise comparisons of estimated marginal means were used to explore within- and between-group effects.

**Exploratory Analyses: VR Session-Level Effects**

We also analyzed secondary outcomes collected only in the treatment group to assess immediate effects of the VR mindfulness sessions. These included scores on the Flow Short Scale (FSS) and brief mood ratings collected pre- and post-session in three monitored sessions. Paired-sample *t*-tests were used to assess changes within each session, and repeated-measures analyses examined whether pre-to-post improvements (e.g., in tension or calmness) changed across sessions, indicating possible cumulative effects. These exploratory analyses were interpreted cautiously due to the smaller sample size.

In summary, the analysis strategy was designed to test the main hypotheses that the FloatVR mindfulness intervention would lead to greater reductions in aggression and emotional distress in the treatment group compared to the control group, while also documenting the immediate experiential effects of the VR sessions on flow and mood. This comprehensive analytical approach ensures that both longer-term intervention outcomes and short-term session effects are rigorously evaluated.

**Linear Mixed-Effects Model Results for Aggression Outcomes**

| **Variable** | **Time × Group Interaction (F, p)** | **Simple Effects (T1, T2, T3) Treatment vs. Control** | **Within-Group Changes (Treatment)** | **Within-Group Changes (Control)** | **Marginal R²** | **Conditional R²** |
| --- | --- | --- | --- | --- | --- | --- |
| Aggression – Total | F(2, 75.668) = 5.972,  p = .004 | T1: ns;  T2: p < .001  T3: p < .001 | T1–T2:  p = .013;  T1–T3:  p < .001 | ns | .220 | .660 |
| Physical Aggression | F(2, 76.619) = 3.132  p = .049 | T1: ns  T2: p = .014  T3: p = .016 | T1–T2:  p = .024  T1–T3:  p = .003 | ns | .154 | .630 |
| Anger | F(2, 75.832) = 2.831  p = .065 | T1: ns  T2: p = .001  T3: p = .002 | T1–T2: ns;  T1–T3:  p = .003 | ns | .186 | .728 |
| Verbal Aggression | F(2, 67.167) = 3.052  p = .054 | T1: ns  T2: p = .004 T3: p = .004 | T1–T2:  p = .053  T1–T3: ns | ns | .198 | .704 |
| Hostility | F(2, 74.963) = 4.654  p = .012 | T1: ns  T2: p = .002 T3: p = .063 | T1–T3:  p = .002 | T1–T2:  p = .00  T2–T3: ns | .125 | .745 |

**Note:** **Note.** Estimated effects of the VR-based mindfulness intervention on total aggression and its subscales (verbal aggression, physical aggression, anger, and hostility) across three time points (T1–T3), controlling for facility. The intervention effect on total aggression was η² = 0.08. Verbal aggression estimates should be interpreted with caution due to a relatively high proportion of missing values.

| **Variable** | **Time × Group Interaction (F, p)** | **Simple Effects (T1, T2, T3) Treatment vs. Control** | **Within-Group Changes (Treatment)** | **Within-Group Changes (Control)** | **Marginal R²** | **Conditional R²** |
| --- | --- | --- | --- | --- | --- | --- |
| DASS Total | F(2, 78.178) = 4.395, p = .016 | T1:  p = .237 T2:  p = .012* T3:  p < .001* | T1 vs. T2:  p = .002*  T1 vs. T3: p < .001* | ns | 0.222 | 0.639 |
| Stress | F(2, 80.38) = 2.621, p = .079 | T1:  p = .098 T2:  p = .010* T3:  p < .001* | T1 vs. T2:  p = .018*  T1 vs. T3:  p < .001* | ns | 0.238 | 0.534 |
| Depression | F(2, 78.54) = 1.903, p = .156 | T1:  p = .379 T2:  p = .055 T3:  p = .008* | T1 vs. T2:  p = .008*  T1 vs. T3:  p < .001* | ns | 0.168 | 0.61 |
| Anxiety | F(2, 79.53) = 5.063, p = .009 | T1:  p = .586 T2:  p = .046* T3:  p < .001* | T1 vs. T2:  p = .019*  T1 vs. T3:  p = .002* | ns | 0.154 | 0.593 |

**Linear Mixed-Effects Model Results for Emotional Distress (DASS-21)
Note:** Estimated effects of the VR-based mindfulness intervention on total emotional distress (DASS) and its subscales (depression, anxiety, and stress) across three time points (T1–T3), with baseline differences adjusted through covariate modeling. η² = 0.10
